# Supplementary material for: Multi‐dataset identification of innovative feature genes and molecular mechanisms in keratoconus
Source: J Cell Mol Med. 2024 Sep 19;28(18):e70079. doi: 10.1111/jcmm.70079 (PMC11412914; doi:10.1111/jcmm.70079)
Supplement: Supplementary file 2 — Material S2. [file JCMM-28-e70079-s002.zip › 7.GSEA analysis/1.ARL11/KEGG_VASCULAR_SMOOTH_MUSCLE_CONTRACTION.html]

Details for gene set KEGG\_VASCULAR\_SMOOTH\_MUSCLE\_CONTRACTION[GSEA]

|  || Dataset | ARL11.group.cls#h\_versus\_l |
| Phenotype | group.cls#h\_versus\_l |
| Upregulated in class | l |
| GeneSet | KEGG\_VASCULAR\_SMOOTH\_MUSCLE\_CONTRACTION |
| Enrichment Score (ES) | -0.2988535 |
| Normalized Enrichment Score (NES) | -1.0620446 |
| Nominal p-value | 0.3600823 |
| FDR q-value | 1.0 |
| FWER p-Value | 0.998 |
Table: GSEA Results Summary

  

Fig 1: Enrichment plot: KEGG\_VASCULAR\_SMOOTH\_MUSCLE\_CONTRACTION      
 Profile of the Running ES Score & Positions of GeneSet Members on the Rank Ordered List

  

| SYMBOL | TITLE | RANK IN GENE LIST | RANK METRIC SCORE | RUNNING ES | CORE ENRICHMENT || 1 | PLCB2 | na | 134 | 0.619 | 0.0482 | No |
| 2 | ARHGEF12 | na | 1468 | 0.340 | 0.0383 | No |
| 3 | CACNA1F | na | 1554 | 0.333 | 0.0638 | No |
| 4 | RAMP1 | na | 1818 | 0.310 | 0.0823 | No |
| 5 | PRKCQ | na | 2774 | 0.251 | 0.0759 | No |
| 6 | CALML6 | na | 3001 | 0.240 | 0.0896 | No |
| 7 | PLA2G4E | na | 4751 | 0.174 | 0.0536 | No |
| 8 | ADCY3 | na | 5276 | 0.158 | 0.0518 | No |
| 9 | ADCY2 | na | 5416 | 0.154 | 0.0607 | No |
| 10 | ADCY9 | na | 5439 | 0.153 | 0.0730 | No |
| 11 | ADCY7 | na | 5559 | 0.149 | 0.0821 | No |
| 12 | CYP4A11 | na | 6286 | 0.129 | 0.0720 | No |
| 13 | PLA2G3 | na | 6310 | 0.129 | 0.0822 | No |
| 14 | MYLK | na | 6649 | 0.120 | 0.0826 | No |
| 15 | ADCY1 | na | 7855 | 0.095 | 0.0558 | No |
| 16 | ADRA1D | na | 8030 | 0.092 | 0.0585 | No |
| 17 | MYH11 | na | 8489 | 0.083 | 0.0522 | No |
| 18 | PLA2G12B | na | 9215 | 0.072 | 0.0373 | No |
| 19 | PRKX | na | 9610 | 0.066 | 0.0315 | No |
| 20 | ROCK1 | na | 9633 | 0.065 | 0.0363 | No |
| 21 | ITPR2 | na | 9655 | 0.065 | 0.0412 | No |
| 22 | ITPR1 | na | 10204 | 0.058 | 0.0302 | No |
| 23 | PLCB3 | na | 10228 | 0.057 | 0.0344 | No |
| 24 | ADCY4 | na | 10290 | 0.056 | 0.0373 | No |
| 25 | ADCY6 | na | 10456 | 0.054 | 0.0371 | No |
| 26 | PRKCA | na | 10617 | 0.052 | 0.0369 | No |
| 27 | BRAF | na | 10628 | 0.052 | 0.0410 | No |
| 28 | KCNMB1 | na | 11471 | 0.042 | 0.0202 | No |
| 29 | CALCRL | na | 11955 | 0.037 | 0.0093 | No |
| 30 | KCNMB2 | na | 12740 | 0.029 | -0.0108 | No |
| 31 | PLA2G2C | na | 13424 | 0.024 | -0.0286 | No |
| 32 | ADORA2B | na | 13467 | 0.023 | -0.0279 | No |
| 33 | PPP1CB | na | 13974 | 0.019 | -0.0409 | No |
| 34 | KCNMA1 | na | 14068 | 0.018 | -0.0420 | No |
| 35 | RAMP3 | na | 14117 | 0.018 | -0.0419 | No |
| 36 | ARHGEF11 | na | 14134 | 0.018 | -0.0409 | No |
| 37 | JMJD7-PLA2G4B | na | 14509 | 0.015 | -0.0504 | No |
| 38 | CALM2 | na | 14606 | 0.014 | -0.0520 | No |
| 39 | ROCK2 | na | 15225 | 0.009 | -0.0691 | No |
| 40 | PPP1R12B | na | 15428 | 0.008 | -0.0742 | No |
| 41 | GNA11 | na | 15711 | 0.006 | -0.0819 | No |
| 42 | GNAS | na | 16257 | 0.002 | -0.0975 | No |
| 43 | PPP1R14A | na | 16497 | 0.000 | -0.1044 | No |
| 44 | ADCY5 | na | 16504 | 0.000 | -0.1045 | No |
| 45 | PPP1CA | na | 16505 | 0.000 | -0.1045 | No |
| 46 | PRKCD | na | 16865 | -0.002 | -0.1147 | No |
| 47 | MAP2K2 | na | 16997 | -0.003 | -0.1182 | No |
| 48 | CALML5 | na | 17116 | -0.004 | -0.1213 | No |
| 49 | PLA2G4A | na | 17245 | -0.005 | -0.1245 | No |
| 50 | RAMP2 | na | 17278 | -0.005 | -0.1250 | No |
| 51 | ADORA2A | na | 17446 | -0.006 | -0.1293 | No |
| 52 | PRKCG | na | 17545 | -0.007 | -0.1316 | No |
| 53 | PLA2G6 | na | 17692 | -0.008 | -0.1351 | No |
| 54 | PLA2G12A | na | 17822 | -0.009 | -0.1380 | No |
| 55 | GNAQ | na | 17828 | -0.009 | -0.1374 | No |
| 56 | ITPR3 | na | 18064 | -0.011 | -0.1433 | No |
| 57 | PLCB4 | na | 18133 | -0.011 | -0.1443 | No |
| 58 | MYL6 | na | 18604 | -0.014 | -0.1567 | No |
| 59 | CALML3 | na | 18646 | -0.015 | -0.1566 | No |
| 60 | ADRA1A | na | 18705 | -0.015 | -0.1571 | No |
| 61 | CALM1 | na | 18762 | -0.015 | -0.1574 | No |
| 62 | PRKACB | na | 18969 | -0.017 | -0.1619 | No |
| 63 | NPR1 | na | 19221 | -0.018 | -0.1676 | No |
| 64 | CACNA1D | na | 19640 | -0.021 | -0.1779 | No |
| 65 | ACTA2 | na | 19755 | -0.022 | -0.1793 | No |
| 66 | MYL6B | na | 19790 | -0.022 | -0.1784 | No |
| 67 | PLA2G2E | na | 19951 | -0.023 | -0.1811 | No |
| 68 | PPP1R12A | na | 20040 | -0.024 | -0.1816 | No |
| 69 | PRKCH | na | 21082 | -0.032 | -0.2090 | No |
| 70 | RHOA | na | 21886 | -0.038 | -0.2290 | No |
| 71 | MAPK3 | na | 22614 | -0.046 | -0.2461 | No |
| 72 | PPP1CC | na | 22638 | -0.047 | -0.2428 | No |
| 73 | CALM3 | na | 22971 | -0.050 | -0.2482 | No |
| 74 | RAF1 | na | 23336 | -0.054 | -0.2542 | No |
| 75 | PRKACA | na | 23665 | -0.058 | -0.2588 | No |
| 76 | PLA2G10 | na | 23788 | -0.060 | -0.2573 | No |
| 77 | CALD1 | na | 23912 | -0.061 | -0.2557 | No |
| 78 | MYL9 | na | 24535 | -0.070 | -0.2678 | No |
| 79 | MAPK1 | na | 24626 | -0.071 | -0.2644 | No |
| 80 | CACNA1C | na | 25293 | -0.080 | -0.2769 | No |
| 81 | GNA12 | na | 25331 | -0.080 | -0.2712 | No |
| 82 | PRKACG | na | 25752 | -0.087 | -0.2761 | No |
| 83 | NPR2 | na | 25877 | -0.088 | -0.2723 | No |
| 84 | ADCY8 | na | 25957 | -0.090 | -0.2670 | No |
| 85 | KCNMB4 | na | 26168 | -0.093 | -0.2653 | No |
| 86 | ARAF | na | 26195 | -0.093 | -0.2581 | No |
| 87 | GNA13 | na | 26206 | -0.094 | -0.2506 | No |
| 88 | KCNMB3 | na | 27297 | -0.115 | -0.2724 | No |
| 89 | MYLK2 | na | 27619 | -0.121 | -0.2715 | No |
| 90 | CYP4A22 | na | 27781 | -0.125 | -0.2656 | No |
| 91 | PLA2G2D | na | 27985 | -0.130 | -0.2605 | No |
| 92 | ARHGEF1 | na | 28229 | -0.137 | -0.2559 | No |
| 93 | AVPR1B | na | 28803 | -0.152 | -0.2597 | No |
| 94 | ADRA1B | na | 29083 | -0.159 | -0.2544 | No |
| 95 | PLA2G2F | na | 30623 | -0.208 | -0.2813 | Yes |
| 96 | AVPR1A | na | 30851 | -0.216 | -0.2697 | Yes |
| 97 | MAP2K1 | na | 31070 | -0.223 | -0.2573 | Yes |
| 98 | GUCY1A2 | na | 31473 | -0.238 | -0.2488 | Yes |
| 99 | PTGIR | na | 31874 | -0.255 | -0.2389 | Yes |
| 100 | AGTR1 | na | 32018 | -0.261 | -0.2211 | Yes |
| 101 | PLA2G5 | na | 32093 | -0.264 | -0.2010 | Yes |
| 102 | ACTG2 | na | 32223 | -0.270 | -0.1820 | Yes |
| 103 | PRKCE | na | 32224 | -0.270 | -0.1593 | Yes |
| 104 | PLCB1 | na | 32251 | -0.272 | -0.1371 | Yes |
| 105 | PLA2G2A | na | 32310 | -0.275 | -0.1156 | Yes |
| 106 | MYLK3 | na | 32788 | -0.307 | -0.1036 | Yes |
| 107 | EDNRA | na | 33087 | -0.328 | -0.0846 | Yes |
| 108 | PLA2G1B | na | 33526 | -0.362 | -0.0668 | Yes |
| 109 | PRKCB | na | 33863 | -0.399 | -0.0430 | Yes |
| 110 | CACNA1S | na | 33887 | -0.403 | -0.0097 | Yes |
| 111 | PRKG1 | na | 33894 | -0.404 | 0.0241 | Yes |
Table: GSEA details [plain text format]

  

Fig 2: KEGG\_VASCULAR\_SMOOTH\_MUSCLE\_CONTRACTION      
 Blue-Pink O' Gram in the Space of the Analyzed GeneSet

  

Fig 3: KEGG\_VASCULAR\_SMOOTH\_MUSCLE\_CONTRACTION: Random ES distribution      
 Gene set null distribution of ES for **KEGG\_VASCULAR\_SMOOTH\_MUSCLE\_CONTRACTION**
